# Commonly Overlooked Factors in Biocompatibility Studies of Neural Implants

**DOI:** 10.1002/advs.202205095

**Published:** 2023-01-03

**Authors:** Lucas S. Kumosa

**Affiliations:** ^1^ Neuronano Research Center Department of Experimental Medical Science Medical Faculty Lund University Medicon Village, Byggnad 404 A2, Scheelevägen 8 Lund 223 81 Sweden

**Keywords:** biocompatibility, neural implants, neural interfaces

## Abstract

Biocompatibility of cutting‐edge neural implants, surgical tools and techniques, and therapeutic technologies is a challenging concept that can be easily misjudged. For example, neural interfaces are routinely gauged on how effectively they determine active neurons near their recording sites. Tissue integration and toxicity of neural interfaces are frequently assessed histologically in animal models to determine tissue morphological and cellular changes in response to surgical implantation and chronic presence. A disconnect between histological and efficacious biocompatibility exists, however, as neuronal numbers frequently observed near electrodes do not match recorded neuronal spiking activity. The downstream effects of the myriad surgical and experimental factors involved in such studies are rarely examined when deciding whether a technology or surgical process is biocompatible. Such surgical factors as anesthesia, temperature excursions, bleed incidence, mechanical forces generated, and metabolic conditions are known to have strong systemic and thus local cellular and extracellular consequences. Many tissue markers are extremely sensitive to the physiological state of cells and tissues, thus significantly impacting histological accuracy. This review aims to shed light on commonly overlooked factors that can have a strong impact on the assessment of neural biocompatibility and to address the mismatch between results stemming from functional and histological methods.

## Graphical abstract

Biocompatibility of cutting‐edge neural implants, surgical tools and techniques, and therapeutic technologies is a challenging concept that can be easily misjudged. This review aims to shed light on commonly overlooked factors that can have a strong impact on the assessment of neural biocompatibility including surgical, experimental, histological, and analytical techniques. By addressing these factors, closer alignment between functional and histological performance is expected, resulting in improved assessments of biocompatibility.

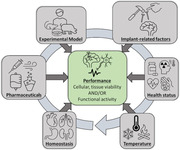

## 1 Introduction

### 1.1 Neural Implant Technology

An aging population,^[^ ^1^, ^2^ ^]^ increased recognition of traumatic brain injuries (TBI) from sports and dangerous professional or recreational activities,^[^ ^3^, ^4^ ^]^ poor health due to “western” lifestyles,^[^ ^5^, ^6^ ^]^ are increasing the prevalence of neurodegenerative disorders. Our continued better understanding of brain structure, function, and health is improving the number of potential strategies to combat neurodegenerative diseases, however, clinically effective therapeutics remain scarce.^[^ ^1^ ^]^ On the forefront, neural interfaces (NI) are receiving much attention as advancements are made in our ability to communicate with the brain, provide therapeutic benefits, and restore damaged functionality.^[^ ^7^, ^8^, ^9^, ^10^, ^11^, ^12^, ^13^, ^14^, ^15^, ^16^, ^17^ ^]^ Recent commercial activity in the field^[^ ^18^, ^19^, ^20^, ^21^ ^]^ has further stoked significant research and development.

Electrical activity in the brain can be measured and manipulated at numerous locations relative to active neuronal populations.^[^ ^22^, ^23^, ^24^, ^25^ ^]^ Non‐invasive scalp surface electroencephalogram (EEG) recording/transcranial stimulation (TCS) can be performed to record and stimulate activity of the cortical layers spatially averaged over a broad area (cm scale). In order to improve spatial resolution, electrodes can be surgically placed intracranially on the surface of the cortical dura (epidural) or directly on the cortex (subdural) to reduce signal averaging over a more localized neuronal population (mm scale), referred to as electrocorticography (ECoG). To measure and stimulate neuronal activity of individual cells (µm scale) or to communicate with specific locations in the subcortical brain, penetrative interfaces of various dimensions and configurations are utilized. Analogs of these technologies have been employed throughout the broader nervous system. Neural activity can also be assessed using numerous non‐electrical modalities, including ultrasonic, optical, chemical, thermal, magnetic, etc., however, each of these modalities ultimately aims to infer and/or manipulate the inherent electrical signaling within nervous tissues.^[^ ^16^, ^17^, ^26^, ^27^, ^28^ ^]^

To introduce such technologies into appropriate locations within the heterogenous brain requires an invasive and penetrative approach. As a result, surgical and insertion damage disrupts the native structure of the brain which may already be highly degenerated through age or disease. The wound healing process and its associated phenomena (localized hemorrhage, inflammation, remodeling, stable interface formation, etc.) are important effectors of neurotechnological success (details of this process are reviewed elsewhere^[^ ^29^, ^30^, ^31^ ^]^). Success can be gauged functionally (does the technology provide the necessary benefit over a therapeutically relevant time course?) but is more commonly assessed in conjunction with histological analysis of interfacial tissues (do wound‐healing phenomena subside resulting in a healthy interface reminiscent of naïve tissues?). Because human neurological disorders are difficult to accurately replicate in animal models,^[^ ^32^ ^]^ and the effects of age and disease are challenging to separate from technological functional failure, histologically determined tissue health is therefore commonly used as a proxy for biocompatibility.

Measures of neural tissue electrical activity by ECoG and EEG represent spatially averaged activity of hundreds to tens of thousands of cells, respectively, resulting in area‐specific low frequency (<250–500 Hz) oscillations referred to as local field potential (LFP).^[^ ^25^ ^]^ By placing microscale electrodes in immediate vicinity of neurons, signals gain high‐frequency spiking patterns which can be separated from underlying, broader, tissue‐specific LFP signals. Spiking activity that cannot be discerned into individual neuronal action potential (AP) waveforms^[^ ^33^ ^]^ is referred to as multiunit spikes (MUs). Quality of parenchymal neural interface recordings is routinely quantified as unique neuronal firing AP waveforms per active electrode (referred to as single units (SUs)) per unit time. This favored metric of microelectrode‐based NI quality relies on stable positioning, healthy surrounding tissues, and maintained electrode quality.^[^ ^34^, ^35^ ^]^ As a result, penetrative NIs that minimally disrupt their surrounding tissues through mechanically matching the stiffness of neural tissues,^[^ ^8^, ^10^, ^16^, ^36^, ^37^, ^38^, ^39^, ^40^ ^]^ avoiding fixation to the skull,^[^ ^41^, ^42^ ^]^ matching density and shape of neural structures,^[^ ^9^, ^42^, ^43^ ^]^ and utilizing insertion techniques that protect underlying tissues,^[^ ^44^, ^45^, ^46^, ^47^, ^48^ ^]^ are prevalent in the research field. Even still, reported SU numbers are far below the expected activity from available neurons within the recording radius of a microelectrode (assumed to be ≈50 µm).^[^ ^49^, ^50^, ^51^ ^]^ Immunohistochemical (IHC) analysis of interfacial tissues adjacent to implanted chronic probes with poor signal recording frequently illustrates that substantial neuronal populations are present within this recording distance.^[^ ^49^ ^]^ Factors that dictate NI efficacy, other than just neuronal presence near active electrodes, must therefore exist. Our recent work presents a unique metabolic and morphological perspective, where visible neurons near such interfaces might not be entirely viable and many larger‐diameter neurons capable of producing stronger signals might be preferentially lost.^[^ ^44^ ^]^ Such findings have motivated this examination of the myriad other factors that might have been overlooked in biocompatibility studies of neural implants which report good biocompatibility based on histology yet fail to provide adequate long‐term efficacy.

### 1.2 Commonly Ascribed Failure Mechanisms

NI failures can be classified in several ways that depend on the point of view of the reviewer (device versus tissue associated, obvious versus non‐obvious failures, healthy versus unhealthy implant tissue, etc.). Failure of the device can be ascribed to material failures such as insulation layer delamination, electrode surface oxidation/degradation, mechanical buckling or dislodgement, connectivity issues, etc.^[^ ^30^, ^34^, ^52^, ^53^, ^54^ ^]^ These are primarily due to manufacturing errors, experimental mishaps, or the harsh conditions found within brain tissues (saline, inflammatory, degradative microenvironments). Tissue‐associated failures are commonly ascribed to excessive inflammatory reactions (gliotic encapsulation, fibrous deposition, reactive oxygen/nitrogen species (RONS) damage, etc.), damage to connective elements (demyelination, axon/dendrite damage, loss of synaptic connectivity), excessive bleeding and vascular disruption, and combinations thereof that ultimately lead to necrotic/apoptotic loss of neurons.^[^ ^30^ ^]^ Non‐obvious failures are more challenging to define. As already mentioned, even when no failure of the device and apparent healthy tissue interface are found, poor recording of neuronal spiking is encountered.^[^ ^49^ ^]^ This is in stark contrast to apparently degraded electrodes and damaged insulation layer, which are associated with glial upregulation, fibrotic deposition, and commensurate changes in electrode impedance that still manage to provide surprisingly adequate neural signal recording.^[^ ^52^ ^]^ Lastly, most biocompatibility research and development are performed using animal models, primarily in young individuals from healthy strains, but in specific cases, disease models are utilized (Parkinsonian animals (pharmacologically lesioned), epileptic mice, stroke‐like ischemic lesioned individuals, etc.).^[^ ^15^, ^16^, ^55^ ^]^ Clinical applications can be segregated between patients with healthy brain tissues but loss of functionality through localized denervation due to injury or disease,^[^ ^35^, ^56^, ^57^ ^]^ or extensively diseased brain tissues (Parkinson's, Alzheimer's, multiple sclerosis, etc.).^[^ ^58^, ^59^, ^60^ ^]^ Limited efficacy of a device to provide adequate therapeutic benefit may not be entirely due to device fault or anomalous tissue healing; overall advanced degenerated state of the tissue, incomplete or poor targeting/stimulation of affected areas, progressive loss of signal as disease state advances, or combinations thereof may dominate.^[^ ^61^ ^]^

### 1.3 Study Aspects that Affect Biocompatibility Studies

There is little consensus in the field about how to surgically implant materials for the purposes of biocompatibility research using animal models. Surgical methods applied to human patients cannot be accurately replicated in animals (a patient can usually be asked to sit still and interacted with during a procedure whereas an animal subject cannot). Surgical variables like anesthesia (choice of anesthetic, dosing, postoperative pain management), temperature regulation (necessity, application method, mode of tracking), insertion procedures (speed of insertion, implant shape, materials and textures, generated forces, location), type of device (epidural, subdural, intracortical), exhibit considerable variability in the literature and therefore direct comparisons between studies are difficult. There is also a high degree of variability in histological results where similar studies reach markedly different conclusions. For example, Freire et al.,^[^ ^62^ ^]^ Jiang et al.,^[^ ^16^ ^]^ and Grand et al.^[^ ^63^ ^]^ report a relatively minor neuronal loss from penetrative devices in rodents using various electrode configurations, whereas Bérces et al.,^[^ ^64^ ^]^ Potter et al.,^[^ ^65^ ^]^ Harris et al.,^[^ ^40^ ^]^ and Stiller et al.^[^ ^21^ ^]^ report noticeable loss within the ≈50 µm recording zone using similar animal models and electrode constructions within sub‐chronic/chronic timeframes. Similarly, glial activity and encapsulation, markers of excessive tissue responses, were found to be visibly higher by Nolta et al.,^[^ ^66^ ^]^ Szarowski et al.,^[^ ^67^ ^]^ and Turner et al.,^[^ ^68^ ^]^ than those found by Freire et al.^[^ ^62^ ^]^ and McCreery et al.^[^ ^69^ ^]^ Bleed incidence and blood‐brain barrier (BBB) leakage, a common consequence of penetrative brain insertions as demonstrated by Nolta et al.,^[^ ^66^ ^]^ Grand et al.,^[^ ^63^ ^]^ Kozai et al.,^[^ ^70^ ^]^ infrequently display comparatively minor disruption to surrounding tissues (Lee et al.^[^ ^37^ ^]^ and Potter et al.^[^ ^65^ ^]^) using similar experimental conditions. Furthermore, each of these markers can be profoundly impacted by homeostatic dysregulation induced by choices of anesthesia, temperature regulation, and insertion process. The situation is compounded by the effects of post‐experimental factors such as euthanasia, tissue processing, histological marker selection, imaging, and analysis.

### 1.4 Aim of this Review

**Figure 1:**
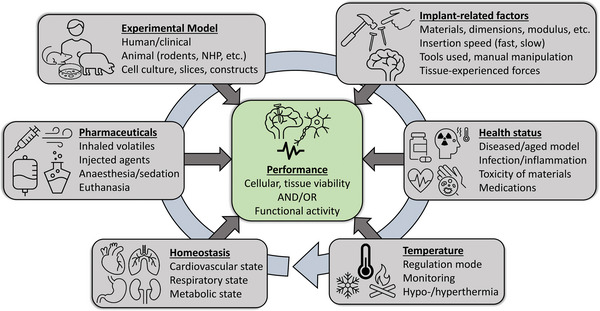
The multivariate complexity of biocompatibility assessment in neural implant research. Study factors such as experimental models (human clinical studies, rodent studies, tissue slices, cell culture models) and health status (healthy, diseased, modified, etc.) dictate what kinds of implant‐related factors can be studied (whole tissue aspects versus cellular/subcellular aspects). Animal models require anesthesia, sedation, and euthanasia protocols that might be unnecessary, unethical, or unfeasible in human studies; such aspects are not applicable to in vitro or ex vivo studies. Pharmaceutical application will have a direct impact on the model organism's homeostasis and regulation including cardiovascular, respiratory, and metabolic disturbances; such homeostasis disruption can reciprocally affect pharmaceutical metabolism and toxicity. A key indicator of homeostasis disruption is body temperature which in most cases needs to be monitored and regulated. Tissue temperature, for example, impacts the mechanical characteristics of tissues which can affect the mechanical aspects of implant insertion and behavior. Moreover, the degree to which homeostasis can be disrupted differs between human studies (minimal homeostasis disruption is appropriate) compared to animal studies (homeostasis disruption is a common consequence of study aspects), and cell culture or tissue slices where homeostasis is drastically altered or abolished altogether. Each of these interrelated factors can have a direct consequence on the organism's health status which might reciprocally affect each of these surgical and experimental factors. Biocompatibility is determined by functional or histological performance (see text for explanation), which can be both significantly and, of importance for this review, disparately affected by the surgical and experimental factors presented. This review focuses on select aspects of neural implant research that appear to be routinely overlooked in the literature when assessing biocompatibility.

This review focuses on the rarely discussed interactions between the surgical/experimental conditions used in biocompatibility studies of neural technologies, the downstream biomolecular alterations as cells/tissues respond, and the resultant IHC and functional analyses used to gauge biocompatibility (see **Figure** **1**). Such interactions may inadvertently misrepresent interfacial tissue quality (both over/under‐estimation of tissue decline are possible) through standard signal recording and IHC methods. It is not the intention of this manuscript to present a complete in‐depth review of each of the many interrelated subjects; merely to propose a line of reasoning for the poor correlation between performance of neural technologies and the histological analysis of tissues that interface with these technologies. In particular, materials used to construct neural implants are an important consideration for how an implant will behave over the long‐term, however, material aspects are extensively studied; material considerations have recently been reviewed by Wellman et al.^[^ ^54^ ^]^ and are therefore not specifically covered in this review. Penetrative NI studies will be the primary focus of this review due to their ability to directly communicate with neural networks thus providing a metric for functional efficacy, their provisional clinical implementation, and similar wound healing‐related phenomena that undermine other technologies which involve penetrative surgical implementation (cell implantations,^[^ ^71^, ^72^, ^73^, ^74^, ^75^, ^76^ ^]^ biopsy collection,^[^ ^77^, ^78^, ^79^ ^]^ microdialysis and drug delivery,^[^ ^17^, ^29^ ^]^ tissue‐engineered construct implantation,^[^ ^80^ ^]^ to name a few).

## 2 Surgical Aspects that Might Impact Subsequent Biocompatibility and Analysis

### 2.1 Anesthesia

In an animal model‐based research setting, systemic anesthesia and stereotaxic fixation are required to surgically implant precision neural devices or cells into the delicate brain. Human procedures rely on some degree of local anesthesia and likely sedatives and muscle relaxants, particularly if dyskinesia is present;^[^ ^72^, ^81^ ^]^ human patients can restrain themselves and verbal interaction with the patient is beneficial in tracking implantation progress, however, general anesthesia may be necessary for more advanced disease states or due to surgical anxiety. Even still, local pharmacological interventions can have systemic effects.^[^ ^82^ ^]^ Of importance are compounds that disrupt homeostasis of temperature, heart rate, respiration and blood/tissue oxygenation, water retention, blood and tissue glucose levels, blood pH, blood pressure, and carbon dioxide levels, to name a few. Each of these phenomena could have effects on surgical efficacy, subsequent wound healing and inflammatory sequelae, and ultimately on device performance and tissue viability.

#### 2.1.1 Homeostasis Varies with Anesthesia Type

**Figure 2:**
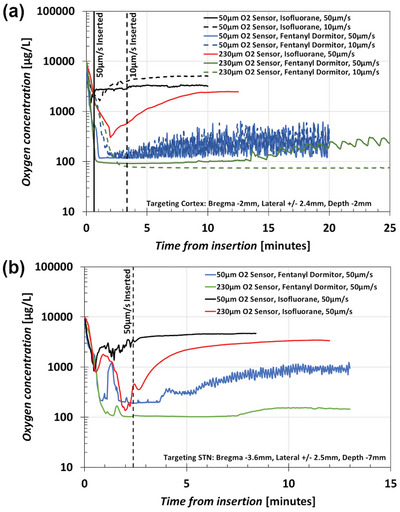
Acute oxygenation during and immediately following transient insertion of oxygen sensors into rat brain using different anesthetics (isoflurane inhalation versus fentanyl/dormitor vet intraperitoneal administration), different insertion speeds, and different tip geometries (230 µm flat cut versus <50 µm diameter rounded tip with 10° taper); *n* = 3 for each condition. Previously unpublished pilot study data used comparatively to select experimental parameters should not be used to gauge absolute oxygen values in the brain. a) Cerebral cortex and b) subthalamic nucleus (STN, only 50 µm s^−1^ speed used due to depth). The transients in (b) between 30 and 120 s represent traversal through the lateral ventricle; cerebral‐spinal fluid infiltration of the target site may therefore overestimate oxygen levels at target. Isoflurane levels maintained between 1.4 – 1.8%; administered fentanyl (0.3 mg kg^−1^ body weight)/dormitor vet (medetomidine hydrochloride, 0.3 mg kg^−1^ body weight). Oxygen concentrations are presented on a logarithmic scale to aid in visualizing separation between the different study conditions. Such data were collected using equipment and surgical procedures consistent with those presented in Kumosa et al., 2018^[^ ^123^ ^]^ and Kumosa & Schouenborg, 2021,^[^ ^44^ ^]^ unless otherwise specified.

In a laboratory setting, anesthesia is applied in such a fashion as to ensure complete sedation of the subject in order to perform required surgical and experimental procedures without interruption. For inhalation anesthetics, this has been defined as minimum alveolar concentration (minimum concentration of volatile drug in lung alveoli that results in complete sedation in 50% of the population of a species, defined as 1 unit MAC), allowing for a useful comparison of sedative effects of different inhaled anesthetic compounds.^[^ ^83^ ^]^ Inhalation anesthetics are generally believed to exhibit less homeostasis disruption than injected barbiturate and opioid forms.^[^ ^83^, ^84^, ^85^, ^86^ ^]^ Intravenous/intraperitoneal/intramuscular administration is more challenging as blood/tissue partitioning, accumulation, and metabolism are complex, requiring specific induction and maintenance dosing with frequent blood level tracking and dose adjustment. No standard dosing among various injected agents equivalent to MAC is currently available to allow comparison; as such expertise and a careful review of the literature are required.^[^ ^87^, ^88^, ^89^ ^]^ When working with small rodents, assisted ventilation is generally not compatible with chronic studies as a high likelihood of damage to the respiratory tract requires ethical euthanasia before anesthesia is terminated. Use of anesthetics that disrupt oxygenation is therefore likely to have a greater impact on tissue viability, as the biomolecular effects of hypoxia on tissue health can be significant.^[^ ^90^, ^91^ ^]^ This is more likely to affect small animal (i.e., rodent) models, as larger animals can be more readily ventilated. Rodent studies examining oxygenation of surgically intruded brain tissues show strong interdependence between anesthesia type (opioid vs inhalation) and resultant oxygen levels in tissues surrounding an inserted oxygen probe, as shown in **Figure** **2** (previously unpublished pilot study data). Beginning in a thin PBS fluid layer on the surface of the exposed cortex, oxygen probe penetration induces a sharp, rapid decline in measured oxygen as the probe descends, followed by recovery to a plateau for the <50‐µm diameter tip probes in isoflurane (IF) anesthetized animals, but not in the fentanyl/dormitor vet (FD) anesthetized animals. Note that the acute effects of anesthesia greatly exceed the effects of probe dimensions and insertion speed (which correlate to the forces exerted on the tissues^[^ ^47^, ^92^ ^]^). For flat‐cut, 230‐µm diameter probes (similar to a microinjection needle), oxygen levels slowly recover using IF, and anoxia can last over 25 min using FD. Most neural interface implantations and pharmaceutical delivery are completed within this timeframe, and such conditions are unlikely to promote neuronal viability. In our prior work using IF,^[^ ^44^ ^]^ we determined that the initial plateau is heavily determined by compressive effects, as slight retraction is immediately followed by an increase in oxygen, however, there is a second longer‐term recovery that occurs (≈90 min) equilibrating with the ventricles. It is not known whether FD anesthesia will follow a similar recovery trend, or if other forms of anesthesia will behave in a manner closer to that of IF or FD, and this would appear to be of importance to the field. While much work is spent on device designs (shapes, materials, mechanics, etc.) and insertion methods (speeds, vehicles, etc.), the effects of anesthetic choice can dominate the tissue environment; thus, subtle differences that might have affected downstream tissue response (e.g., see the effects on neuronal survival and gliosis in Kumosa & Schouenborg, 2021^[^ ^44^ ^]^) may be lost due to severe hypoxic stress during surgery.

#### 2.1.2 Dosage Effects

Proper dosage can be as important as the choice of anesthetic itself. Insulin dysregulation has been found when using inhalation anesthetics in humans, resulting in reduced blood glucose levels and increased norepinephrine levels, compared to pre‐anesthesia controls and patients immobilized using spinal tap anesthesia administration.^[^ ^93^ ^]^ Interestingly, increased glucose levels were found under higher dosages despite similarly increased insulin and cortisol levels (0.035 — 1.125 MAC halothane vs 1.3 MAC isoflurane, respectively) suggesting metabolic compromise.^[^ ^94^ ^]^ Moreover, cortisol levels have direct effects on blood flow rates, blood pressure, and therefore risk of hemorrhage and BBB disruption, as discussed later.

When examining studies in the literature that utilized inhalation anesthetics, reported doses higher than required (2 — 3 MAC to induce, 1.5 — 2 MAC during surgery, and 1.2 — 1.5 MAC during experiment) are commonly employed.^[^ ^21^, ^45^, ^52^, ^65^, ^92^, ^95^, ^96^, ^97^, ^98^, ^99^, ^100^, ^101^, ^102^, ^103^, ^104^, ^105^, ^106^ ^]^ By carefully maintaining sedation at the minimally required isoflurane dosage (0.8 — 1 MAC), improved neural activity can be monitored using cortical EEG. By raising isoflurane levels to those commonly used in small animal research, neural activity is suppressed.^[^ ^107^, ^108^ ^]^ A similar effect has been demonstrated using a vascular stent‐mounted ECoG‐type electrode array^[^ ^19^ ^]^ and a parenchymal implanted NI.^[^ ^16^ ^]^ Not all stimuli have the same impact on an anesthetized subject. In dogs, for example, spontaneous movement was found to require less anesthetic to maintain sedation (0.46% halothane) than insertion of endotracheal tube (0.55%), skin incision (0.69%), or mild electric shock (10 volts, 0.66%), and both >30 s tail clamp and moderate (30 volts)/severe (50 volts) electric shock required the highest anesthetic doses (0.81 – 0.85%, equivalent to ≈1 MAC).^[^ ^83^ ^]^ It should also be noted that stimulus tolerance under anesthesia differs across species and changes with age,^[^ ^109^ ^]^ and even intraspecies variations such as hair color are recognized to influence efficacious anesthesia dosage.^[^ ^110^ ^]^ Lastly, since MAC levels are not deterministic of sedation levels in any given subject, animal‐to‐animal variability can be expected.

Animal feeding state has also been implicated in anesthesia efficacy, demonstrating that well‐fed animals (likely with higher blood glucose levels and correspondingly increased insulin levels) require higher levels of anesthesia to attain optimal sedation, thus risking fatal consequences.^[^ ^111^ ^]^ Such increased anesthesia could further disrupt homeostasis and interfere with neural activity, thereby exacerbating the multitude of issues raised in this review. Animal studies are frequently performed on animals fed ad libitum, or with no mention of feeding status ahead of surgery. Human surgical intervention is either performed under local anesthesia as mentioned, or if general anesthesia is to be used patients are instructed not to eat or drink the day before surgery.

### 2.2 Body Temperature

Most systemic anesthetics will disrupt homeostasis to some degree, where unless controlled, loss of body temperature regulation can be expected. The effects of temperature on neural wound healing and tissue health can be quite pronounced. Even slight temperature deviations (2–3 °C) can greatly impact tissue function and stress response where hypothermia has long been known to provide neuroprotection during and immediately following surgical interventions and brain trauma.^[^ ^112^, ^113^, ^114^ ^]^ It is commonly assumed that the cause for this is the suppression of neural tissue metabolism.^[^ ^114^ ^]^ However, hypothermia's impact on numerous cell stress response pathways has been identified.^[^ ^112^ ^]^ Careful examination of microdialysis perfusates collected during ischemia/reperfusion (IR) models of stroke damage have shown that when brain tissue temperature is properly maintained, the brain displays strong amino acid neurotransmitter (glutamate, dopamine, aspartate, glycine) disturbances that can be quite long‐lasting.^[^ ^115^, ^116^, ^117^ ^]^ Such disturbance is presumed to make *N*‐methyl‐D‐aspartate‐type glutamate receptors hyperexcitable potentially contributing to neuronal excitatory damage. Such disturbances can be envisioned as altering intracellular amino acid pools available for protein production. When slight hypothermia is applied, neurotransmitter disturbances are mitigated, and tissues generally display less histopathological damage despite maintaining similar metabolic profiles (glucose, glycogen, lactate, pyruvate, adenosine phosphate levels).^[^ ^118^ ^]^ This is corroborated by findings of general neural activity suppression under hypothermic conditions.^[^ ^119^ ^]^ Furthermore, fatty acid stores in brain structures are altered from IR injuries; hypothermia is shown to favorably modulate such lipid alterations.^[^ ^115^ ^]^ Moreover, hypothermia was demonstrated to reduce inflammatory sequelae by inhibiting leukocyte adhesion and transmigration signals^[^ ^120^ ^]^ and reducing BBB permeability by inhibiting basal lamina degradation following ischemia.^[^ ^121^ ^]^ For completeness, it should be mentioned that hyperthermia is a frequent consequence of disease, infection, and inflammation, localized hyperthermia has been reported in cases of electrical and optogenetic stimulation, and investigated as a mode of neural suppression,^[^ ^28^, ^122^ ^]^ however mild hyperthermic stress has been found to compromise neuronal resilience^[^ ^118^ ^]^ and caution is advised.

#### 2.2.1 External Temperature Regulation

**Figure 3:**
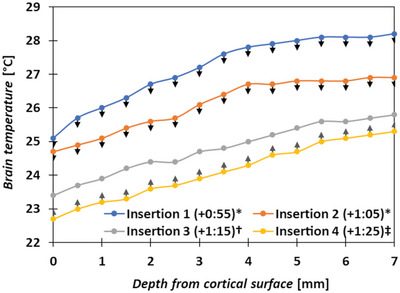
Pilot study temperature measurements in rat brain performed under fentanyl/dormitory vet anesthesia (administered intraperitoneally at *t* = 0) with no core body temperature regulation, however, all measurements were performed before animal's core temperature fell below 32 °C (monitored using a rectal probe). Brain measurements were performed using a 250 µm‐diameter, tapered tip probe (MT‐29/3, AgnThos AB, Sweden) inserted/removed at 50 µm s^−1^ speed; temperatures were recorded every 0.5 mm during descent, and changes were noted upon subsequent ascent. Note the rapid (≈1 °C per 10 min) drop in brain temperature between the insertions and the strong depth gradient (0.37 °C mm^−1^). Insertions were performed in independent locations (± 2.4 mm from midline, −2 mm and −6 mm from bregma, 1 = left rostral, 2 = right rostral, 3 = left caudal, and 4 = right caudal). Interestingly, the animal's brain temperature regulation appeared active as the first two insertions displayed 0.2–0.3 °C lower temperatures upon removal of probe (*), the next insertion showed no temperature difference upon probe removal (†), and the last insertion showed temperatures 0.2–0.3 °C higher upon probe removal (‡). Animal was euthanized upon completion of final measurement. Details of surgical/experimental procedures can be found in Kumosa et al., 2018.^[^ ^123^ ^]^

Heat application to the body core with rectal probe monitoring is commonly employed to regulate a rodent's body temperature, assuming a commensurate effect in the brain. This however has been shown to be misleading, where even during such heating, brain temperatures can be considerably lower than the core;^[^ ^118^ ^]^ therefore, it is safe to assume that most small animal neural biocompatibility studies have been performed under some measure of cerebral hypothermia. In our previous works we employed two forms of anesthesia: intraperitoneal fentanyl/dormitor vet with no heat regulation,^[^ ^123^ ^]^ and inhaled isoflurane with 37 °C heat‐pad core heating via rectal probe control.^[^ ^44^ ^]^ Pilot study data (see **Figure** **3**) revealed that in the former, brain temperature freefall is present, and strong depth‐dependent gradients in the small rodent brain exist. When animals were heated under isoflurane anesthesia, cortical temperatures were still >5 °C lower than the body core (31.7 ± 0.7 °C at 1.5 mm depth using a 250 µm diameter tapered‐tip temperature probe in cortex; *n* = 10), albeit stable. Choice of anesthetic is also critical where propofol anesthesia under normothermic conditions appears to convey some measure of amino acid neurotransmitter‐regulating neuroprotection similar to mild hypothermia.^[^ ^117^ ^]^ As mentioned earlier, human NI implantations are commonly performed under surgical conditions that minimize homeostasis disruption. In order to maintain more physiological brain temperatures in small animal studies, use of a targeted heat lamp and control through a local intramuscular (temporalis muscle) temperature probe as described by Busto et al.^[^ ^118^ ^]^ is recommended.

### 2.3 Bleeding Incidence and Sequalae

At present time, cortical implants in the research setting are routinely inserted by merely observing the brain surface vasculature, inferring the predominantly vertical arrangement of underlying larger vessel structures, and hoping not to “hit anything important” during device descent. High variability in blood‐borne immunoglobulin gamma (IgG) staining seen in IHC follow‐up within 2 h of cortical stab injury suggests however that reliable prediction cannot be made about vascular structures below the surface.^[^ ^123^ ^]^ Longer implants that target deep structures such as the subthalamic nucleus, habenula, etc. could potentially employ preoperative imaging (and in some cases during the surgical procedure) such as magnetic resonance imaging (MRI), computed tomography (CT) modalities, laser speckle imaging (LSI), or high‐resolution ultrasound to select an appropriate trajectory.^[^ ^12^, ^124^, ^125^ ^]^ Such trajectory selection is commonplace in clinical implantations. However, even such powerful imaging modalities only allow for avoiding larger vessels; due to their extreme density and compounded by cardiovascular and respiratory motions,^[^ ^30^, ^126^, ^127^ ^]^ meso‐ and microvessels cannot be effectively avoided.

Damage to vascular structures during device implantation has been well documented, illustrating the rupture of vessels, dragging of vascular tissues with an implant into the depths of the brain, and fluid buildup and leakage that results.^[^ ^128^ ^]^ Assessment of the tissue bleeding that results from implant insertion is routinely reported in the literature and presents a wealth of information regarding subsequent wound healing dynamics.^[^ ^29^, ^52^, ^65^, ^66^, ^129^ ^]^ Larger implants or implants with multiple protruding elements generally produce more bleed damage. The negative consequences of bleeding surrounding NIs have been extensively documented, both from implant degradation^[^ ^52^, ^66^ ^]^ and tissue inflammation^[^ ^29^, ^65^, ^129^, ^130^ ^]^ perspectives. We performed a comprehensive analysis of wound healing linked to insertion speed, implant size, and use of gelatin coating to abate the effects of vessel rupture^[^ ^123^ ^]^ illustrating the detrimental effects of BBB leakage following transient device insertion (250 µm‐diameter needles, inserted slowly at 10–50 µm s^−1^). In that study, while the effects of prolonged BBB disruption were clear in terms of increased IgG) leakage and heightened inflammatory glial activation, a high degree of variability was observed. In a few rare cases, there appeared to be no readily apparent bleed or only minimal post‐insertion inflammation even in cases of uncoated implants inserted at higher velocities. A high degree of local heterogeneity of neural tissues must therefore exist for such cases to occur.

#### 2.3.1 Clinical Studies of Bleeding Incidence

Clinical studies illustrate real‐world bleed incidence and consequences. Biopsy collection of brain tissues can provide critical benefits for identifying complex neurological disorders such as oncological sub‐cortical lesions^[^ ^77^, ^78^, ^131^ ^]^ and dementia.^[^ ^79^, ^132^ ^]^ While preoperative imaging to select an appropriate site of biopsy is routine, postoperative imaging is performed for patients that demonstrate a symptomatic change upon follow‐up or if excessive bleeding is observed (or suspected) during the biopsy collection procedure. As such, it is commonly believed that <10% of patients experience bleeding from biopsy collections. However, not all bleeds are symptomatic, and patients suffering from pathological neurodegeneration are frequently not cognizant of neurological changes.^[^ ^133^, ^134^ ^]^ In a study that performed postoperative imaging of the biopsy site irrespective of patient follow‐up performance, it was found that ≈60% of patients developed some level of biopsy‐induced hemorrhage within several hours as visible using CT (mm‐scale resolution).^[^ ^131^ ^]^ It should be mentioned that a similar study found lower rates (7%) when imaging was performed within 2 h and found highest prevalence of bleeds in brainstem (31%).^[^ ^78^ ^]^ Hemorrhage occurrence may thus be time delayed, resolution dependent, brain‐structure specific, or population variable. Side‐cutting needle biopsy collection is similar in dimensions (≈1.5 mm diameter^[^ ^135^ ^]^) to current clinical neural stimulation technology (e.g., deep brain stimulation (DBS) electrodes).^[^ ^136^ ^]^ This suggests that brain hemorrhage induced by or following surgical device insertion can be suspected, and in most cases is asymptomatic.

In clinical applications, DBS implantation involves a multi‐stage process assisted by a temporary cannula inserted ahead of the DBS electrode, and either removed before final electrode placement creating an insertion track, or after the electrode is placed using the canula as a guide. Even still, the reported incidence of hemorrhage during post‐operative imaging (MRI and/or CT imaging with mm‐scale resolution performed 4 to 72 h following surgery, hematoma volume greater than 0.2 cm^3^ considered) was found to be ≈3%.^[^ ^58^, ^137^, ^138^ ^]^ While the placed device likely acts as a plug within disrupted vasculature, given the predilection for BBB leakage seen in chronic microelectrode interface biocompatibility studies,^[^ ^65^, ^66^, ^130^ ^]^ this clinical DBS hemorrhage incidence rate seems perplexingly low. It should be noted that post‐implantation MRI can be complicated by metallic components which can interfere with imaging near the implant interface. Also, the leakage of the BBB and bleeding are not equivalent, where even if not ruptured, mechanically induced dynamic changes to the BBB^[^ ^139^ ^]^ may allow certain plasma constituents to infiltrate even if RBC‐containing whole blood does not. The act of removal may be bleed‐inducing, which represents a potential concern for transient device insertions such as cell transplants and microinjections of pharmaceuticals.

#### 2.3.2 Study‐Related Inducers of Bleeding and Vascular Disruption

Given the likelihood of poor outcomes following bleeding events in the brain (reminiscent of hemorrhagic stroke),^[^ ^29^ ^]^ or the significant effects on wound healing that excessive insertion‐induced damage can have,^[^ ^52^, ^66^, ^67^ ^]^ it is surprising that studies are frequently performed using manually inserted instruments.^[^ ^66^, ^95^, ^96^, ^97^, ^98^, ^99^, ^140^, ^141^ ^]^ Moreover, there is no agreement on what tool is used to surgically induce an experimental wound, where scalpel blades, hypodermic needles, functional and non‐functional NI constructions, along with multitude of custom‐designed shapes have been used. Each of these would likely generate different forces, insertion damage, and resultant bleeding, and therefore would induce varying levels of inflammatory response. For example, it has been suggested that a sharp, lower diameter implant would benefit from insertion at higher speeds that prevent deformations of soft tissues to restrict damage to only the “slicing” damage due to the implant.^[^ ^46^, ^92^, ^128^ ^]^ Larger implants, with dull, rounded tips however might benefit from slower speeds to provide delicate tissue structures (microvessels, cells) time to rearrange near the advancing implant surface^[^ ^92^, ^128^ ^]^ and avoid crushing damage. These two insertion strategies can be envisioned to produce substantially different wound‐healing responses. Speed of intracortical microelectrode insertion has been linked to recording performance and acute neuronal survival by Fiáth et al.^[^ ^48^ ^]^ Notably, apart from our two recent studies^[^ ^44^, ^45^ ^]^ and the detailed work of Welkenhuysen et al.,^[^ ^47^ ^]^ correlation of force measurements during insertions with subsequent histological follow‐up to correlate wound healing progression is largely absent in the literature.

### 2.4 Mechanical Forces Experienced by Tissues

Many studies have measured the macroscopic^[^ ^92^, ^142^, ^143^, ^144^ ^]^ and implant‐localized mechanical properties^[^ ^44^, ^45^, ^47^, ^92^, ^100^, ^101^, ^145^, ^146^, ^147^, ^148^ ^]^ of brain tissue. Studies by Schouenborg et al., for example, have shown that the mechanical forces that are experienced by the living brain during insertion and around an implant can have significant wound healing and metabolic implications for brain tissues.^[^ ^36^, ^37^, ^42^, ^43^, ^44^, ^45^, ^123^ ^]^ The mechanical properties of the brain are also known to change with temperature, where anesthesia and body‐core heating‐related hypothermia can cause stiffening of the brain.^[^ ^149^ ^]^ Confoundingly, the mechanical properties of the brain itself are also spatially heterogeneous and dynamic during aging and disease.^[^ ^150^, ^151^ ^]^ By inserting rigid implants into the brain, reducing micromotion‐induced damage by avoiding anchorage, or by reducing implant density, both of which are believed to reduce the forces experienced by tissues, have beneficial effects by reducing gliosis.^[^ ^42^, ^43^ ^]^ By reducing speed of insertion, and therefore likely the experienced forces,^[^ ^92^ ^]^ we showed that insertion‐induced BBB leakage and subsequent inflammatory sequelae were reduced.^[^ ^123^ ^]^ Curiously, nanostructured polycrystalline silicon implant surfaces, which would be intuited to generate more surface area‐induced friction during insertion and thus require greater forces compared with smooth surface counterparts, result in slightly reduced glial encapsulation and improved neuronal survival,^[^ ^64^ ^]^ possibly due to the anti‐wetting “lotus effect”.^[^ ^152^ ^]^

The brain is almost entirely enclosed in a hard skull and suspended in a cerebral‐spinal fluid that provides some measure of force dissipation. Therefore, it might seem surprising that the brain has mechanical sensation capabilities.^[^ ^27^ ^]^ Astrocytes, the primary glial cell population within the brain, have been shown to be exquisitely sensitive to mechanical disturbances, both in vitro^[^ ^102^, ^153^, ^154^ ^]^ and in vivo.^[^ ^8^, ^42^, ^43^, ^44^, ^123^ ^]^ As an important component of the neurovascular unit, astrocytes maintain “communication” between the vasculature and neighboring neurons;^[^ ^139^, ^155^, ^156^ ^]^ mechanical disturbance may thus modulate their neurovascular coupling. Moreover, the delicate structures of the brain surface where the pia matter has extensive astrocyte process density, are easily intruded upon or inadvertently damaged during delicate surgical procedures when preparing for neural implantation or insertion. In a prior study, we found insertion injury‐induced upregulation of astrocytes and gelatinases (matrix metalloproteinases (MMP) 2 and 9) that are believed to play a role in BBB integrity, where magnitude of astrocyte upregulation depended on speed of insertion (and therefore tissue‐experienced force).^[^ ^123^ ^]^ MMPs have been previously linked to mechanical activation of both microglia and astrocytes.^[^ ^102^ ^]^

Mechanical sensitivity by neurons has also been demonstrated, however, the exact implications are unknown.^[^ ^27^, ^157^ ^]^ The soma can withstand some compressive disturbance^[^ ^42^, ^43^, ^123^, ^158^ ^]^ as evidenced by histological examination, however, reports indicate that mechanical disruption can significantly alter neuronal spike dynamics.^[^ ^157^, ^159^ ^]^ As early as 1970, Humphrey suggested that improved neural SU recording performance using individually advanceable microelectrode arrays could be achieved when insertion‐induced tissue compression was allowed to resolve before final electrode positioning.^[^ ^160^ ^]^ Whether neurons are susceptible to low‐level mechanical stress is unresolved where both resilience to indentation^[^ ^157^ ^]^ and susceptibility to stretching^[^ ^102^ ^]^ have been demonstrated in vitro. Stress and strain‐associated tissue deformations, such as those likely induced by implanting larger protruding microwire arrays, particularly at higher insertion speeds, have been likened to TBI and result in altered neural activity.^[^ ^161^, ^162^ ^]^ Interestingly, by inserting a flexible device that reduces such tissue‐experienced forces, and further orienting mode of flexion in the direction of predominant brain motions (fore/aft),^[^ ^163^ ^]^ significant benefits for both inflammation and neuronal survival were found.^[^ ^8^ ^]^ Our recent works investigating the forces of rigid probe insertion on neuronal survival show a clear benefit of reduced mechanical disruption in vivo and therefore improved tissue health by devising insertion methods that alleviate insertion‐induced forces, by either coating in low‐friction ice‐coated gelatin,^[^ ^45^ ^]^ or by introducing a slight retraction after passing implant target depth.^[^ ^44^ ^]^

There has been limited research into the interactions between interfaces and non‐somal neuronal components. It is unclear what effects repeated motions against a slim, flexible, yet ultimately stronger micron‐sized metallic/polymeric construct will have on small and delicate neuronal and glial projections; slicing, demyelination, synaptic uncoupling, microvessel compression, can be easily envisioned.^[^ ^29^ ^]^ Depending on force of impact on neuronal compartments (soma, axons, dendrites) different calcium influx responses have been demonstrated in vitro, namely transient responses resolved in seconds or sustained responses lasting minutes.^[^ ^157^ ^]^ Such transients might significantly alter activity and spike waveforms as metal ion gradients are disturbed.^[^ ^33^ ^]^ Myelination dynamics have been recently studied in detail illustrating biphasic demyelination‐remyelination patterns surrounding implanted NIs,^[^ ^164^ ^]^ however there does not appear to be a strong correlation with signal recording quality.^[^ ^165^ ^]^ Further work addressing the interaction between neural implants and neuronal projections is needed.

## 3 Histological Variability due to Physiological States

Most assessments of biocompatibility in neural interfacial tissues adjacent to implants are made based on histological analysis where interfacial tissue morphology is compared to an appropriate control. This is usually performed ex vivo after an animal is euthanized at the end of an experimental period, but in some instances is also performed in vivo using live imaging (2‐photon, radiation imaging, etc.). In a clinical setting, such analysis is only performed postmortem. The most commonly evaluated cell types are neurons and glia (astrocytes and microglia/macrophages),^[^ ^24^, ^30^ ^]^ with oligodendrocytes, endothelial cells, pericytes, invading leukocytes, etc. being investigated less frequently.^[^ ^166^ ^]^ Epitopes used to identify these cell types have been discovered either empirically as in the case of neuronal marker NeuN,^[^ ^167^ ^]^ or by happenstance, where a marker is first identified in other cell types, tissues, animal models, such as the case of Iba1 used in microglial/macrophage identification.^[^ ^168^ ^]^

**Figure 4:**
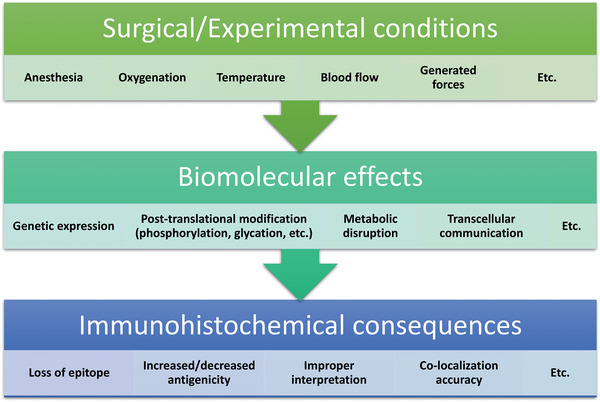
Impact of surgical/experimental conditions on the bio‐molecular processes in cells and tissues that can directly impact IHC assessment of biocompatibility.

As stated earlier, the surgical and/or experimental conditions to which the subject is exposed induce significant biomolecular effects both at the local cellular and tissue level, but also potentially at the systemic level (see **Figure** **4**). These biomolecular effects can be expected to impact the biomolecular structures (proteins, nucleic acids, glycosaminoglycans, etc.) that are used as epitopes for histological analysis. A few of the more ubiquitous stains will be discussed as examples of the complications that can arise.

### 3.1 Neuronal Identification using NeuN

**Figure 5:**
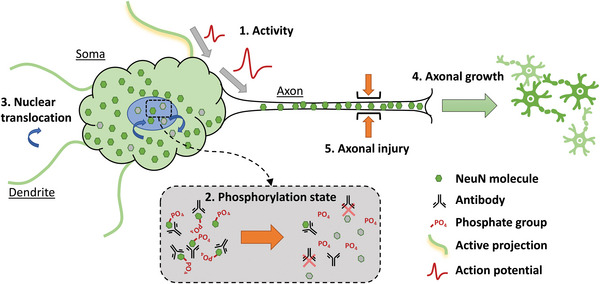
Schematic representation of NeuN staining variability dependent on neuronal state. Identified modulators of NeuN antigenicity that could affect neuronal identification and quantification include 1) activity,^[^ ^173^ ^]^ where neurons receiving and transmitting action potentials reduce NeuN expression, 2) physiological state,^[^ ^171^ ^]^ where dephosphorylation of NeuN is believed to interfere with antibody‐epitope binding, 3) cytosolic/nuclear localization,^[^ ^171^ ^]^ where alternative‐splicing isoforms of NeuN been found to differentially undergo nuclear translocation from the cytosol, 4) axonal growth,^[^ ^173^ ^]^ where neurons actively expanding their axonal projections following injury increase their NeuN content, and 5) axonal injury^[^ ^174^ ^]^ can abolish NeuN expression in certain CNS projection neurons. Ischemic hypoxia is also known to interfere with NeuN antigenicity^[^ ^172^ ^]^ where the exact mechanism is unknown, however, cell stress including hyperglycemia, ROS generation, and edema following ischemic reperfusion could disrupt epitope structure by promoting dephosphorylation and protein degradation.

NeuN (neuronal nuclear antigen, also known as Rbfox3, RNA binding protein feminizing locus on X homolog 3) is a marker routinely used to identify mature neurons in the brain.^[^ ^167^ ^]^ The antigenicity of NeuN has been shown to be highly variable (see **Figure** **5**). First empirically determined as a general‐purpose neuronal marker specific to most neuronal populations in the mammalian CNS, NeuN was later identified as an RNA‐binding protein product believed to play a role in mRNA alternative splicing.^[^ ^169^, ^170^ ^]^ NeuN is found both in the nucleus and in neuronal cytosol,^[^ ^171^ ^]^ and it's staining has been reported to be highly susceptible to cellular physiological state, namely phosphorylation modifications^[^ ^171^ ^]^ and ischemic hypoxia.^[^ ^123^, ^172^ ^]^ NeuN expression has also been linked to a neuron's state of axonogenesis and found to down‐regulate under conditions of high neuronal activity or axonal injury.^[^ ^173^, ^174^ ^]^ Recently it has been demonstrated that NeuN antigenicity (along with other neuronal protein markers) is lost immediately following BBB disruption and this loss can be long‐lasting.^[^ ^175^ ^]^ Comparative staining using other methods shows that overall neuronal density is relatively unchanged at sites of disruption, suggesting neurons are alive, and that their protein expression is altered.^[^ ^62^, ^175^ ^]^ Neuronal metabolic viability however might not be retained.^[^ ^44^, ^62^, ^123^ ^]^ Thus, interpretation of NeuN staining in nervous tissues collected from surgically injured animals experiencing a history of variable levels of stress, neuronal activity, and metabolic disturbances (both naturally occurring and surgically/experimentally induced) can be complicated. By combining neuronal identification using immunohistological methods with tracking of endogenous autofluorescent metabolic components, neuronal viability can be assessed.^[^ ^44^ ^]^

### 3.2 GFAP as a Marker of Astrocyte Activity

**Figure 6:**
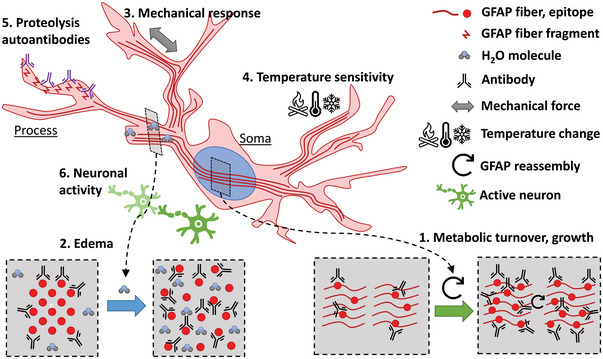
Schematic representation of GFAP staining variability dependent on astrocytic state. Identified modulators of GFAP antigenicity that could affect astrocytic identification and quantification include: 1) metabolic turnover of GFAP filaments exposes previously hidden epitope sites,^[^ ^177^ ^]^ 2) injury or pathological edema formation interrupting fibril packing exposing otherwise hidden epitopes,^[^ ^178^ ^]^ 3) mechanical sensitivity and upregulation of GFAP,^[^ ^44^, ^123^, ^153^ ^]^ 4) temperature sensitivity,^[^ ^182^, ^183^ ^]^ 5) cell‐death induced proteolysis autoantibodies,^[^ ^104^ ^]^ and 6) neuronal activity upregulates GFAP expression in astrocytes.^[^ ^185^ ^]^ This is not an exhaustive list and other inducers of GFAP epitope variability likely exist beyond the scope of this review.

GFAP (glial fibrillary acid protein) is an intermediate filament expressed by astrocytes; its increased intensity in cases of brain trauma is commonly used as a gauge of astrocyte upregulation and a proxy for injury severity.^[^ ^8^, ^10^, ^24^, ^41^, ^42^, ^44^, ^45^, ^47^, ^62^, ^63^, ^129^, ^130^ ^]^ Furthermore, GFAP upregulation can be particularly long‐lasting, where injections of buffered saline demonstrate visible GFAP‐stained tracks one year later.^[^ ^176^ ^]^ However, GFAP staining intensity is highly variable (see **Figure** **6**) and has been reported to not correlate with actual fibril quantity.^[^ ^177^, ^178^ ^]^ Metabolic turnover of amino acids in inflammatory tissues is posited to expose otherwise hidden antigenic epitopes without increasing overall amount of protein.^[^ ^177^ ^]^ Additionally, edema, known to occur immediately after focal injury^[^ ^30^, ^179^ ^]^ and following ischemic and metabolic stress,^[^ ^180^, ^181^ ^]^ is believed to alter the packing of glial intermediate fibrils thereby affecting the availability of epitope sites for antibody labeling.^[^ ^178^ ^]^ As discussed earlier, astrocytes have been found to be highly sensitive to mechanical disturbances, upregulating their GFAP expression as a result.^[^ ^44^, ^123^, ^153^ ^]^ GFAP intensity has also been demonstrated to be highly responsive to temperature excursions with both hyper‐ and hypothermic stress manifesting altered staining.^[^ ^182^, ^183^ ^]^ Reproductive hormonal cycles in females also vary GFAP expression.^[^ ^103^ ^]^ Cell‐death‐related degradation has been found in TBI injuries that result in autoantibodies that target GFAP in astrocytes;^[^ ^104^ ^]^ such autoantibody targeting could conceivably interfere with IHC GFAP identification leading to underestimation of actual GFAP content. Relatedly, astrocyte expression of GFAP has been shown to acutely decline following BBB permeation and blood plasma infiltration into brain parenchyma,^[^ ^184^ ^]^ similar to neuronal protein effects.^[^ ^175^ ^]^ Finally, activity from neighboring neurons has been shown to promote GFAP expression in astrocytes;^[^ ^185^ ^]^ therefore surgical factors such as hypothermia or excessive anesthesia that suppress neuronal activity may consequently suppress astrocytic GFAP content. Again, interpretation of IHC GFAP staining is a complicated task. Improper identification of astrocyte reactivity has recently been addressed by a cadre of neuroscientists,^[^ ^186^ ^]^ where reliance on any individual marker is dissuaded.

### 3.3 Microglial and Macrophage Markers (Iba1 and CD68/ED1)

Iba1 (ionized calcium‐binding adapter molecule 1) is a marker used to identify microglia and macrophages in the brain. Originally identified as a marker associated with rejected cardiac allografts (named allograft inflammatory factor 1 (AIF1)),^[^ ^168^ ^]^ it was later isolated as an insulin‐inducible intestinal factor, and further identified as pancreatic macrophage marker in prediabetic/diabetic rats.^[^ ^187^ ^]^ Coincidently, Iba1 was found to label microglia/macrophages in the brain.^[^ ^188^ ^]^ Consensus on whether Iba1 identifies all microglia/macrophages (M1 + M2) or just activated ones (M1) is lacking.^[^ ^189^ ^]^ Furthermore, its ability to modulate insulin secretion and thus influence glucose metabolism^[^ ^187^ ^]^ suggests that glucose and insulin levels might inversely influence Iba1 expression by microglia/macrophages and thus confound their purported activation state.

CD68 (also known as ED1 in rodents) is a marker of activated (M1) macrophage/microglial lysosome membranes.^[^ ^190^ ^]^ It is expressed in inflamed tissues where microglia enter a state of high metabolism, RONS generation, and phagocytosis/exocytosis to break down damaged tissues and foreign contaminants.^[^ ^191^ ^]^ This process requires a significant metabolic supply, both of glucose and oxygen. Lack of these critical nutrients may lead to abnormally diminished acute microglial activation and be mistakenly construed as beneficial.^[^ ^192^ ^]^ Interestingly, reducing BBB permeability through use of protective gelatin coatings was found to preferentially shift to an Iba1+/ED1‐ microglial phenotype 3 days post‐injury compared to Iba1+/ED1+ cells following bare stainless steel transient insertions.^[^ ^123^ ^]^

The “no‐reflow phenomenon”, where prolonged blood flow interruption due to physical blockade does not immediately restore upon removal of blockage,^[^ ^193^ ^]^ results from a backlog of un‐extravasated polymorphonuclear leukocytes and thrombosis.^[^ ^105^ ^]^ Blood‐borne monocytes are known to infiltrate tissues downstream in post‐capillary bed venules.^[^ ^156^ ^]^ Resultantly, subdued acute inflammation due to impediment of blood‐borne phagocyte recruitment (including Iba1/CD68 positive macrophages) may result in compromised chronic tissue viability as wound healing may be incomplete, particularly in the presence of a chronic implant or TBI (for an example of loss of blood flow near an implanted NI, see Figure 5 of Kozai et al., 2015^[^ ^29^ ^]^). The notion that either excessive *or* insufficient leukocyte‐driven inflammatory oxidative stress may lead to device biocompatibility failures has been posited.^[^ ^192^ ^]^ In line with this suggestion, incomplete inflammatory action has been suspected as a possible cause of failure of clinical trials investigating pharmaceutical agents that downregulate microglial activity following stroke and TBI.^[^ ^60^, ^194^ ^]^

### 3.4 Blood‐Brain Barrier Disruption Evidenced by Immunoglobulins

**Figure 7:**
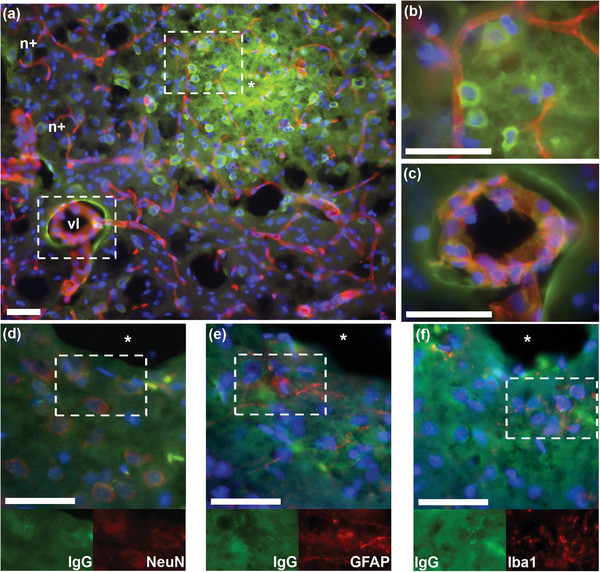
IgG distribution around a site of stab injury in rat cerebral cortex and accumulation in neurons within 2 h of injury. Stab was created using a 250 µm diameter stainless steel needle inserted at 10 µm s^−1^, held in place for 10 min, removed at same speed. a) Stab site imaged at 20× (0.46 µm px^−1^) with high magnification images (100× oil immersion, 0.09 µm px^−1^) of b) neuronal IgG accumulation near undisturbed vessels, and c) vascular leakage distal from stab site. Anti‐rat IgG antibodies (green), RECA: rat endothelial cell antigen (red), and DAPI (blue). Illustrated are the damage‐induced origins of IgG near the stab (*). Of note, there appear to be neurons heavily IgG positive (n+) that are not close to ruptured vessels, and vascular leakage (vl) distal from the stab site. To determine cellular expression, images were captured at 40× (0.22 µm px^−1^) from consecutive slices of a stab site (see Figure 3A, <2‐h SS‐L condition, of Kumosa et al., 2018^[^ ^123^ ^]^) stained with d) IgG (green), NeuN (red), DAPI (blue), e) IgG (green), GFAP (red), DAPI (blue), and f) IgG (green), Iba1 (red), DAPI (blue). Note that the IgG expression appears to strongly coincide with specific neuronal populations (2 of 3 neurons within box in (d) show IgG expression), whereas astrocytes (GFAP (e)) and microglia (Iba1 (f)) do not specifically coincide with IgG deposits. All scale bars = 50 µm. Surgical and histological procedures are detailed in Kumosa et al., 2018.^[^ ^123^ ^]^

IgGs (immunoglobulin gamma, ≈250 kDa) are serum antibodies primarily produced by the body's immune system in response to and subsequent anticipation of foreign biochemical structures. This has led to IgGs becoming the proverbial workhorse of histochemistry by creating uniquely specific antibodies to selected biomolecules. IgGs’ endogenous presence in the blood means they are frequently used to detect abnormal permeation of the BBB.^[^ ^65^, ^123^, ^130^, ^195^ ^]^ However, immunoglobulins are not exclusively produced by immune cells. Brain‐localized IgG production has been reported with both degenerative^[^ ^196^ ^]^ and protective^[^ ^197^ ^]^ roles suggested. In our previous study we observed IgG neuronal accumulation within 2 h of stab injury that appears to be at least partially neuronal in origin (see **Figure** **7** presented here, and Figures 2 and 3 of Kumosa et al., 2018^[^ ^123^ ^]^), where neurons distal from leaking vessels contain IgG.^[^ ^123^ ^]^ Of note is the possibility that trauma‐induced autoantibody production might interfere with identification of intended epitopes.^[^ ^104^ ^]^ Such interference may help explain some of the transient loss of neuronal and astrocytic markers following surgical injury and possibly affect other epitopes as well. Alternative blood‐derived markers of BBB disruption such as fibrinogen (350–420 kDa)^[^ ^198^, ^199^ ^]^ and albumin (65–70 kDa),^[^ ^196^, ^200^, ^201^ ^]^ the latter of which is not presently known to endogenously occur within brain parenchyma, may be used to mitigate some of these uncertainties and discriminate size range of leaked blood constituents.^[^ ^121^ ^]^ Moreover, albumin's role in osmolality regulation, capillary bed fluid balance, protein transport across vascular barriers, and high neuroinflammatory nature, make it an attractive alternative.

### 3.5 Nuclear Chromatin Markers

DAPI (4’,6‐diamidino‐2‐phenylindole) is a fluorescent molecule most utilized for IHC cell nucleus identification in neural biocompatibility studies. Known to integrate itself into nucleic acid structures, its fluorescence is greatly selective to the type of nucleic acid, the base pairs present, and the exact location of attachment.^[^ ^202^, ^203^ ^]^ Specifically, A‐T rich regions of double‐stranded DNA allow DAPI to bind to the minor groove where the molecule becomes highly fluorescent at 365/480 nm wavelength (excitation/emission; ultraviolet/blue). Intercalating at sites other than the minor groove, binding in C‐G rich regions or binding with RNA impedes fluorescence. However, DAPI is known to alter its fluorescence signature under specific intracellular conditions: excessive phosphorylation,^[^ ^204^ ^]^ regions of dense RNA,^[^ ^202^ ^]^ and formation of calcium phosphate^[^ ^204^ ^]^ can promote DAPI binding and red‐shift its fluorescence; high DAPI concentrations exacerbate these effects. Nuclear accumulation of calcium is known to occur following cellular stress.^[^ ^205^, ^206^, ^207^ ^]^ Prolonged UV light exposure will further alter excitation/emission characteristics of DAPI and related Hoechst^©^ variants.^[^ ^208^ ^]^ Such effects could conceivably interfere with identification of cytosolic/nuclear epitopes using a chromatically close dye (e.g., fluorescein derivatives). Alternative nuclear markers such as propidium iodide, ethidium bromide, YOYO/TOTO all provide similar nucleic acid specificity, albeit at varying excitation/emission wavelengths.^[^ ^209^, ^210^ ^]^

### 3.6 Autofluorescent Tissue Features

**Figure 8:**
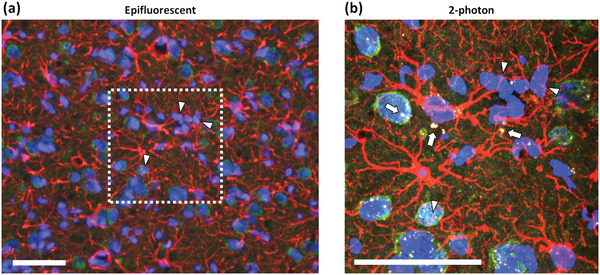
Autofluorescent features in an injured rat cortical tissue slice imaged first using a) epifluorescent imaging and subsequently using b) 2‐photon imaging. Visible bright yellow AF granules in (b) (arrows) that are undetectable in (a) might interfere with epitope quantification. Note that fine features can be identified in both imaging modalities (triangles identify GFAP astrocyte process overlapping a neuronal soma in both (a) and (b)), illustrating that this is a fluorescence/imaging modality limitation. Scale bars = 50 µm. Slides stained with NeuN (green), GFAP (red), and DAPI (blue); details of tissue preparation and imaging can be found in Kumosa & Schouenborg, 2021.^[^ ^44^ ^]^ The 2‐photon image in (b) is a maximum intensity projection through all z‐stack layers that approximates the greater depth‐of‐field imposed by epifluorescent microscopy.

Autofluorescence (AF) of tissues is also a considerable confounder of histological staining analysis, particularly in injured/inflamed tissues.^[^ ^211^ ^]^ It has been long recognized that various tissue structures possess biomolecular components with fluorescent signatures similar to those of fluorescent molecules (fluorescein, rhodamine, intercalating nucleic acid labels, etc.).^[^ ^212^, ^213^, ^214^ ^]^ Commonly recognized AF molecules are lipofuscin and hemosiderin‐laden deposits,^[^ ^211^, ^215^, ^216^ ^]^ metabolic machinery constituents (flavins, nicotinamides, etc.),^[^ ^213^, ^217^, ^218^, ^219^ ^]^ elastic extracellular matrix (ECM) components,^[^ ^213^, ^214^ ^]^ among others. Excessive tissue fixation can further exacerbate AF signals. Methods to combat such AF “contamination” have been employed, including UV bleaching,^[^ ^211^, ^220^ ^]^ lipid dyes (Sudan black, TrueBlack (Biotium, Inc.), etc.),^[^ ^211^, ^221^ ^]^ metal ion quenching,^[^ ^211^, ^216^, ^221^ ^]^ however at the risk of greatly diminishing sensitivity. Therefore, such AF can present a serious challenge, requiring analysis of control tissues with sequential antibody analysis (see Figures S1–S3 (Supporting Information) of Kumosa et al., 2018^[^ ^123^ ^]^ for example workflow). High‐intensity laser scanning methods such as confocal and multiphoton imaging can be extremely sensitive to such AF signals^[^ ^44^, ^218^, ^219^ ^]^ that are not as readily identifiable using epifluorescent imaging (illustrated in **Figure** **8**); such AF itself can however provide powerful insight into tissue health.^[^ ^44^, ^222^ ^]^

### 3.7 Euthanasia and Tissue Post‐Processing

To perform discussed IHC analyses, animals must be euthanized in an ethically responsible manner that preserves tissue structure and protects (as best as possible) epitope antigenicity.^[^ ^223^ ^]^ These techniques frequently utilize systemic molecules such as pentobarbital, CO_2_, ketamine, etc. that do not kill the animal immediately; respiration will usually cease before cardiac activity. This will cause systemic hypoxia and possibly commensurate hyperglycemia. Cell death itself is hard to define^[^ ^224^ ^]^ as different brain cell types can exhibit disparate metabolic rates when exposed to perfusates and anesthetics,^[^ ^225^ ^]^ likely leading to “post‐death” effects. If ethically and practically feasible, cervical dislocation followed by flash freezing might provide a more tissue‐accurate outcome.

Good tissue preservation relies on some form of tissue crosslinking (either in the animal using the vasculature as a delivery route or following excision, a flash freeze, and slicing).^[^ ^226^, ^227^ ^]^ Resultantly, extended immersion in fixatives after tissue excision is commonplace. In many cases, tissue preservation and epitope antigenicity are incompatible requiring subsequent epitope retrieval steps (high temperature and/or chemical agents used to selectively disrupt excessive crosslinking and recover native epitope structure).^[^ ^228^, ^229^ ^]^ Due to excessive background noise, alterations in epitopes, damage to tissue structures, etc., inferring staining quality following epitope retrieval (ER) and obtaining reliable quantitative data is a serious challenge, and research into improved ER methods is ongoing.

### 3.8 Co‐Localization of Multiple Stained Features

Co‐localization of multiple stains is commonly employed in order to minimize uncertainty that arises from any individual staining procedure and to uncover spatial relationships between tissue features. This relies on identifying spatial overlap of epitopes that are known to label a specific cell type or tissue feature.^[^ ^44^, ^55^, ^123^, ^129^, ^140^, ^230^, ^231^ ^]^ However, colocalization analysis is a deceptively complex technique that has limitations. For example, astrocytic process encroachment on neuronal soma can be seen in high‐resolution images.^[^ ^44^ ^]^ In certain cases of stress, astrocytes have been shown to extend processes into neuronal soma cytosol presumably for biomolecular transport.^[^ ^232^, ^233^, ^234^ ^]^ In our recent study, we have further identified astrocytic intrusion upon neuronal nuclei in cases of apparent surgical injury that are readily visible in a sizable proportion of neurons within the injury zone.^[^ ^44^ ^]^ Along with the afore mentioned AF features, such activity could plausibly interfere with colocalization studies (see Figure 8 in this document, and Figure S8 (Supporting Information) of Kumosa & Schouenborg, 2021^[^ ^44^ ^]^) and may confound interpretation. As already discussed, the myriad of metabolic and environmental factors could have a profound impact on the variable expression of these markers.

### 3.9 Prospects for Future Histological Work

Many other antigens used to investigate neural cell types, extracellular and vascular structures, and other proteinaceous targets are likely similarly susceptible to the various environmental, surgical, metabolic, and histopathological conditions discussed so far. It is unrealistic that all permutations be effectively analyzed and therefore a better understanding of the biochemical intricacies that underpin IHC is invaluable for this type of research. Frequently utilized epitopes include neuronal proliferation and migration marker DCX,^[^ ^235^ ^]^ cell cycle markers (e.g., Ki67, BrdU),^[^ ^236^, ^237^ ^]^ cytoskeletal components (e.g., tubulins, actins, intermediate filaments),^[^ ^238^, ^239^ ^]^ ECM constituents (proteoglycans/glycosaminoglycans, collagens, fibronectins, etc.)^[^ ^106^, ^240^, ^241^ ^]^ and would benefit from a similar discussion in the future. Alternatively, labeling of mRNA transcriptional products (e.g., fluorescence in situ hybridization) might provide insight into gene expression,^[^ ^242^ ^]^ however these are subject to posttranscriptional manipulations such as interference and degradation, improper translation and protein folding, etc., and therefore may not directly relate to actual protein quantities. Staining using chemical dyes (hematoxylin and eosin, Nissl stains, trichrome stains, etc.) that does not rely on antibody labeling of epitopes might also assist in identification of tissue structures both chemically and morphologically; however, such stains require extensive histopathological training and usually result in qualitative/semi‐quantitative assessments. Chemical dyes rely on slight differences in pH, water and lipid content, elastic fiber density, and alterations in protein folding,^[^ ^243^ ^]^ and might therefore also be susceptible to surgically and experimentally induced factors, such as various metabolic effects (phosphorylation, glycation, acetylation), edema, etc. Moreover, an analytical eye when evaluating and comparing prior published studies is necessary. Lastly, proper controls, adequate statistical power, and appropriate subsequent analysis are vital to properly interpret results.^[^ ^244^, ^245^ ^]^

## 4 Are all Neurons supposed to be Active?

The IHC inconsistencies raised herein, such as reported neuronal densities that arise from neuronal markers and inconsistent extent of gliotic responses resulting from variable astrocytic and microglial/macrophagic identification, bring us back to the low SU recordings from tissues that appear histologically viable. This issue has been raised since the earliest days of NI design where recorded spikes greatly underestimated the predicted number of neurons within recording distance of the electrode surface.^[^ ^50^, ^51^, ^246^ ^]^ Early hypotheses claimed that insertion‐induced cellular rupture and coating of electrode surfaces with torn cellular membranes, inadvertently placing electrodes inside neighboring glial cells, damage to the electrode surfaces themselves, compression and blockage of limited extracellular electrochemical signal transduction pathways, etc. were responsible for this mismatch.^[^ ^50^ ^]^ This line of reasoning has dominated NI research and design.^[^ ^14^, ^17^, ^22^, ^24^, ^30^ ^]^ A plausible alternative, the “quiet brain” hypothesis, has since been put forth to address this discrepancy. Claiming from a metabolic perspective, even with its immense 20% consumption of available inspired oxygen,^[^ ^247^ ^]^ the human brain cannot feasibly support more than occasional spiking from only a small subset of viable neurons.^[^ ^248^, ^249^ ^]^ The energetics of maintaining and re‐establishing membrane potentials from spiking activity in all neurons at even a modest rate would overwhelm the available oxygen supply of the entire organism, not just the organ itself. On the other hand, the body optimizes the energetic cost of maintaining tissues to ensure adequate utilization (e.g., muscle/bone atrophy in response to lower experienced forces, changes in vascularization in response to metabolic demand dynamics, protective fibrous deposition in response to encountered friction and tissue wear, etc.). This suggests that even if not actively spiking, apparently silent neurons and their extensive axonal and dendritic arborization perform other vital functions that warrant their cost (passive connectivity, trophic support, fast‐acting redundancy, information processing, and storage, etc.). The possibility, therefore exists that low numbers of spikes should be expected, and it is their patterns and transmissible networks that are being disturbed. How extensive this disruption from surgical intrusion into the brain and whether it is reversible requires further investigation.

### 4.1 Other Implantation‐Related Modulators of Neuronal Activity

The compressive mechanical forces that are necessary to insert an implant,^[^ ^44^, ^45^, ^47^, ^100^ ^]^ along with subsequent wound healing tissue cicatricial contraction,^[^ ^250^ ^]^ and micromotions to which chronic implants are subjected,^[^ ^30^, ^126^, ^127^, ^159^ ^]^ may possibly exaggerate the acute signaling that is recorded as neuronal ion channels are known to be susceptible to mechanical activation.^[^ ^27^, ^157^, ^159^ ^]^ Osmolar changes likely occurring following insertion‐induced ischemia/reperfusion events^[^ ^30^, ^179^ ^]^ and subsequent inflammatory phenomena,^[^ ^181^ ^]^ have also been long recognized to modulate frequency and amplitude of neuronal activity.^[^ ^251^, ^252^, ^253^ ^]^ These phenomena are expected to subside over time. We have recently demonstrated that slight differences in insertion forces can have significant effects on neural tissue oxygenation and thus selectively decrease the proportion of larger, likely more powerful, projection neurons one week later.^[^ ^44^ ^]^ Furthermore, the columnar assembly of neocortical neuronal circuits comprising excitatory and inhibitory units with stratified input/output layers^[^ ^254^ ^]^ and the heterogeneously distributed complexity of neuronal interconnectivity^[^ ^255^ ^]^ suggests that depending on depth and location of implantation, neuronal activity at recording locations may be noticeably altered by disruption to affiliated circuitry. Additionally, maintaining stable position of a NI within tissues, particularly those undergoing wound healing responses, is a constant challenge where even slight positional changes can have extreme effects on recording SUs^[^ ^35^, ^246^ ^]^ (it should be reiterated that effective spike recording is limited to within ≈50 µm distance). Some of these phenomena may at first glance present as histologically “normal” and be overlooked. As the implant‐tissue interface stabilizes, decreased neuronal firing rates commonly reported may in part be a normal consequence of healing and in part due to alterations to tissue structure, electrode position, and interface quality resulting from chronic placement; this however requires further investigation.

Most biocompatibility studies are performed in healthy animal models,^[^ ^256^ ^]^ particularly when histological performance is considered. However, recent studies of therapeutic cell transplantations have highlighted subtle differences in responses of neuronal network connections that arise in diseased and aged models even when no overt loss in neuron density or only minor differences in gliosis are noted,^[^ ^75^ ^]^ and disparate connectivity behavior in inflamed and traumatically injured brains.^[^ ^76^ ^]^ How these findings relate to implanted devices, other disease conditions, and human clinical applications remains to be determined. In a clinical study that examined cerebellar morphology of essential tremor patients, those undergoing thalamic DBS preserved synaptic connectivity in their Purkinje cell climbing fibers compared with non‐stimulated patients who displayed a strong correlation between synaptic loss and tremor severity;^[^ ^257^ ^]^ such evidence of wider network impact that extends far beyond the site of implantation suggests that broader considerations of neural biocompatibility be considered. In animal models of Parkinson's and epilepsy,^[^ ^15^, ^16^ ^]^ standard histological evaluations of neuronal disruption and inflammatory gliosis, ubiquitously used in neural implant studies discussed in this review, do not demonstrate notable differences in response to implanted neural interfaces as compared to studies using healthy animals. Encouragingly, preliminary work implanting cutting‐edge, flexible, spatially expanding, multi‐electrode arrays point to long‐term functional biocompatibility in both rodent pain^[^ ^13^ ^]^ and epilepsy^[^ ^16^ ^]^ models. While Schouenborg et al. utilize customizable network targeting specificity in spatially precise pain and movement circuitries,^[^ ^13^, ^15^ ^]^ Jiang et al. demonstrate complementary multifunctional capabilities including optical and chemical modulation of neural tissues.^[^ ^16^ ^]^

Interestingly, chronic indwelling cortical brain‐computer interfaces (Utah arrays comprised of 100 rigid spikes, 1 – 1.5 mm in length, 10 × 10 arrangement at 400 µm pitch) in human and non‐human primate studies that do not rigorously hold to the design guidelines of stiffness matching, automated insertions, micromotion abatement, etc. display chronic performance that can last many years.^[^ ^35^, ^56^, ^57^, ^258^ ^]^ Despite meningeal encapsulation, significant displacement (both out of and into the brain were observed), and a slowly progressive loss of signal, the most common failure mechanisms involved manufacturing errors, connectivity issues, and other non‐biological factors.^[^ ^258^ ^]^ It is likely that these technologies are more resilient to the foreign body response than commonly asserted in the biocompatibility literature. While improvements to the technology are important in advancing therapeutic potential (increasing indwelling time, improving signal content and stability, reducing side effects, reducing need for frequent calibrations, reducing need to replace the sensors, wireless operation, improving patient self‐sufficiency),^[^ ^35^, ^61^ ^]^ these are less likely to be determined by the tissue response.

On the other hand, NIs that communicate with smaller structures located deeper in the brain (subthalamic nucleus, periventricular/periaqueductal gray region, thalamus, hippocampus, etc.)^[^ ^13^, ^15^, ^16^ ^]^ may be more dependent on stable positioning and minimal neuronal disruption that is likely not possible by larger, rigid, multi‐shank constructions. For example, devices such as clinical DBS electrodes used to stimulate large volumes of tissue for successful treatment of Parkinson's and essential tremor symptoms have proven less effective at treatment of depression, chronic pain, and obsessive‐compulsive disorders that likely afflict specific circuits in confined volumes of brain tissue.^[^ ^58^ ^]^ In order to avoid many of the discussed complications of penetrative implants, ECoG communication on the surface of the cortex can be employed.^[^ ^22^, ^23^ ^]^ Novel ultrasoft transparent ECoG arrays, such as those designed by Fedor et al. to minimally irritate the cortical surface, open up concurrent in vivo imaging, and electrophysiological recording capabilities in relatively undisturbed tissues.^[^ ^10^ ^]^ As already mentioned ECoG provides area‐averaged neural activity spanning hundreds to thousands of neurons and does not provide direct information regarding deeper structures.^[^ ^25^ ^]^ Furthermore, such recordings do not provide a direct relationship to neuronal activity, as state of wakefulness, complex dipolar orientation, and both layer and population‐specific activity complicate recorded LFP signals.^[^ ^25^ ^]^ Vascular stent‐mounted ECoG‐style electrode arrays developed by Oxley et al. present a novel approach to address some of these limitations (deep brain access via vasculature, no parenchymal penetration, improved alignment with neural layer dipoles).^[^ ^19^, ^20^ ^]^

Device designs and surgical methods that combine electrical signal recording, chemical analysis, force, and temperature measurement, and blood flow tracking capabilities, to name a few, are likely to become important to better evaluate the immediate tissue health and viability of the implantation site (see examples^[^ ^14^, ^16^, ^17^, ^26^, ^259^ ^]^). Furthermore, surgical interventions should also be chosen in such a manner as to better compliment the experimental questions of interest. For example, impact of anesthesia dosage on neuronal activity was described in Section 2.1.2, however, choice of anesthetic can also affect neuronal signaling^[^ ^260^ ^]^ which likely influences acute recordings; it remains to be determined whether sustained neuronal activity during acute surgery, implantation, and initial inflammatory cascade are beneficial to long‐term performance of neural implants or whether subdued activity would be preferable. Biocompatibility analysis of technologies such as tissue‐engineered constructs, cell implantations, microdialysis collections, pharmaceutical injections, etc.^[^ ^29^, ^71^, ^72^, ^73^, ^74^, ^77^, ^78^, ^79^, ^80^ ^]^ may be similarly confounded by the biological responses discussed in this review. Along with corresponding re‐examination of IHC protocols and imaging analysis/interpretation, these modifications to future neural implant studies are expected to improve alignment between functional and histological biocompatibility.

## 5 Conclusion

Lack of expected efficacy of implantable neural technologies in the aim of treating traumatic and neurodegenerative states has proved to be of considerable consternation to researchers. The significant societal burden that brain injury and neurodegenerative diseases present, both in terms of economic costs and societal burden, implores that, new perspectives are presented to advance research and design efforts of interventions aimed at tackling these debilitating conditions. Lastly, many commercial endeavors that aim at bringing such interventions to the clinic likely lack the time and focus to meaningfully investigate all the factors raised herein. This review is therefore presented to shed light on factors that are routinely overlooked in the neural biocompatibility literature that may otherwise keep future studies from attaining their needed clinical impact.

## Conflict of Interest

The author declares no conflict of interest.
